# Minimally invasive right colectomy with transrectal natural orifice extraction: could this be the next step forward?

**DOI:** 10.1007/s10151-020-02282-x

**Published:** 2020-07-06

**Authors:** C.-C. Cheng, Y.-R. Hsu, Y.-J. Chern, W.-S. Tsai, H.-Y. Hung, C.-K. Liao, J.-M. Chiang, P.-S. Hsieh, J.-F. You

**Affiliations:** 1 Division of Colon and Rectal Surgery, Chang Gung Memorial Hospital, Linkou, Chang Gung University College of Medicine, No. 5, Fu-Hsing St., Kuei-Shan, Taoyuan, Taiwan; 2 School of Medicine, Chang Gung University, Taoyuan, Taiwan

**Keywords:** Minimally invasive surgery, Right colectomy, Right hemicolectomy, Natural orifice specimen extraction (NOSE), Laparoscopic surgery

## Abstract

**Background:**

The transvaginal natural orifice specimen extraction (NOSE) approach for right-side colon surgery has been proven to exhibit favorable short-term outcomes. However, thus far, no study has reported the advantages of transrectal NOSE for right-side colon surgery. The aim of this study was to compare the technical feasibility, safety, and short-term outcomes of minimally invasive right hemicolectomy using the transrectal NOSE method and those of conventional mini-laparotomy specimen extraction.

**Methods:**

A study was conducted on consecutive patients who had minimally invasive right hemicolectomy either for malignancy or benign disease at Chang Gung Memorial Hospital, Linkou, Taiwan, between January 2017 and December 2018. The patients were divided into two groups: conventional surgery with specimen extraction using mini-laparotomy and NOSE surgery. Surgical outcomes, including complications, postoperative short-term recovery, and pain intensity, were analyzed.

**Results:**

We enrolled 297 patients (151 males, mean age 64.9 ± 12.8 years) who had minimally invasive right hemicolectomy. Of these 297 patients, 272 patients had conventional surgery with specimen extraction through mini-laparotomy and 25 patients had NOSE surgery (23 transrectal, 2 transvaginal). The diagnosis of colon disease did not differ significantly between the conventional and NOSE groups. Postoperative morbidity and mortality rates were comparable. The postoperative hospital stay was significantly (*p* = 0.004) shorter in the NOSE group (median 5 days, range 3–17 days) than in the conventional group (median 7 days, range 3–45 days). Postoperative pain was significantly (*p* = 0.026 on postoperative day 1 and *p* = 0.002 on postoperative day 2) greater in the conventional group than in the NOSE group.

**Conclusions:**

NOSE was associated with acceptable short-term surgical outcomes that were comparable to those of conventional surgery. NOSE results in less postoperative wound pain and a shorter hospital stay than conventional surgery. Larger studies are needed

## Introduction

Minimally invasive surgery for colorectal disease is a global trend. Over the past 30 years, minimally invasive colorectal surgery has been shown to cause less postoperative pain, earlier return of bowel function, shorter hospital stays, and fewer wound complications than open surgery [1, 2]. In addition to providing more favorable short-term outcomes than conventional surgery, the long-term outcomes of minimally invasive colectomy and those of open surgery are comparable [2–6]. However, in colorectal surgery, the specimen is extracted through a mini-laparotomy, which entails a 3–8-cm incision depending on the size of specimen. Abdominal incision wounds may negate many of the benefits of minimally invasive surgery [7]. Thanks to the advances made in minimally invasive colorectal surgery, natural orifice specimen extraction (NOSE) surgery can prevent the mini-laparotomy wound.

NOSE surgery was first published in early 1990s. In 1991 and 1992, reports by Stewart et al. [8] and Nezhat [9], respectively, described the extraction of a colectomy specimen through the vagina. Franklin et al. first described colectomy with specimen extraction through the anus in 1993 [10]. Left-side colectomy by the transrectal NOSE method is safe and feasible in some patients; the short-term outcomes include less postoperative pain and a shorter hospital stay than the conventional method. Furthermore, the long-term oncological outcomes are comparable to those of conventional mini-laparotomy specimen extraction [11–13]. Extracting a specimen of the right-sided colon from the resection wound of the colon using a colonoscope is challenging because of the anatomically narrow and tortuous shape of the sigmoid colon. Eshuis’ case series reported specimen extraction through colotomy; however, the extraction failed in two of ten patients because of the bulk of the specimen [14]. Karagul reported that only approximately two-thirds of the unselected laparoscopic colectomy patients were suitable for NOSE. The success rate of the NOSE method was lower in male than in female patients, and also lower for large than for small tumors [15]. Because of technical difficulty, use of NOSE is limited. Transvaginal NOSE has remained the most commonly used path to extract specimens of the right colon [16–18]. However, transvaginal NOSE is limited to female patients and sexual dysfunction after vaginal incision may cause concern. There is currently no scientific literature on the use of transrectal NOSE for extracting specimens of the right colon.

In this study, the short-term outcomes in patients who had undergone minimally invasive right hemicolectomy by the transrectal NOSE approach and those in patients who underwent the surgery by the conventional mini-laparotomy specimen extraction were compared.

## Materials and methods

### Study design and patient selection

Detailed information regarding clinicopathological variables was retrieved from the Colorectal Section Tumor Registry, a prospectively collected database of colorectal cancer patients in a single medical institute of Chang Gung Memorial Hospital, Taiwan, since 1995. The institutional review board approved this study (IRB no. 201901457B0).

Between January 2017 and December 2018, a total of 303 patients had undergone minimally invasive right hemicolectomy either for malignancy or benign disease. Six patients were excluded because of failed minimally invasive surgery and conversion to laparotomy. The remaining 297 patients, among whom 272 received conventional surgery with specimen extraction through mini-laparotomy and 25 received NOSE surgery, were enrolled in this study. The adoption of NOSE surgery was based on each physician’s preference. However, patient characteristics, including body mass index (BMI) > 35 kg/m^2^, the American Society of Anesthesiologists (ASA) class > III, and tumor diameter of > 4 cm, and T4 substage on clinical computed tomography (CT)scan for malignancies were not selected for NOSE surgery. There was a total of 15 surgeons included in this study, and 4 surgeons performed the NOSE procedures.

### Operative procedures

Minimally invasive right hemicolectomy was performed either by a laparoscopic or robotic approach. The standard technique of performing laparoscopic right hemicolectomy involved the use of four ports. For robotic surgery, the DaVinci Xi system (Intuitive, Sunnyvale, USA) was adopted with four robotic arms. Both laparoscopic and robotic surgery involved a similar medial-to-lateral surgical strategy. In the beginning, the dissection plane was along the ileocolic vessels. The ileocolic vessels were clearly defined and divided at their roots for malignant cases. Then retroperitoneal dissection principally adhered to the methods from medial-to-lateral and bottom-to-up approaches. The plane of dissection was anterior to and upwards along the descending portion of the duodenum, lateral to the ascending colon by separating Toldt’s fascia, and heading in a right superior direction along the plane above Gerota’s fascia as far as possible to the hepatic flexure of the colon. Subsequently, mobilization of the lateral attachment of the bowel, including separation of the omentum, gastrocolic and hepatocolic ligaments, and lateral peritoneal attachment of the ascending colon and lower attachment of the terminal ileum, was performed to prevent tension of the anastomosis.

After complete division of the mesentery including the marginal artery, the ileocolic anastomosis was performed either by extracorporeal anastomosis (EA) or intracorporeal anastomosis (IA). For EA, the right-sided colon and terminal ileum were exteriorized through a midline incision by extending the umbilical port wound. The ileocolic anastomosis was created either by the side-to-side stapler method or end-to-end hand-sew method. For IA, the ends of the transverse colon and terminal ileum were divided using GIA staplers, and the anastomosis was created either by side-to-side stapler anastomosis and the use of sutures to close the resulting opening or by the end-to-side hand-sewn method.

**Fig. 1:**
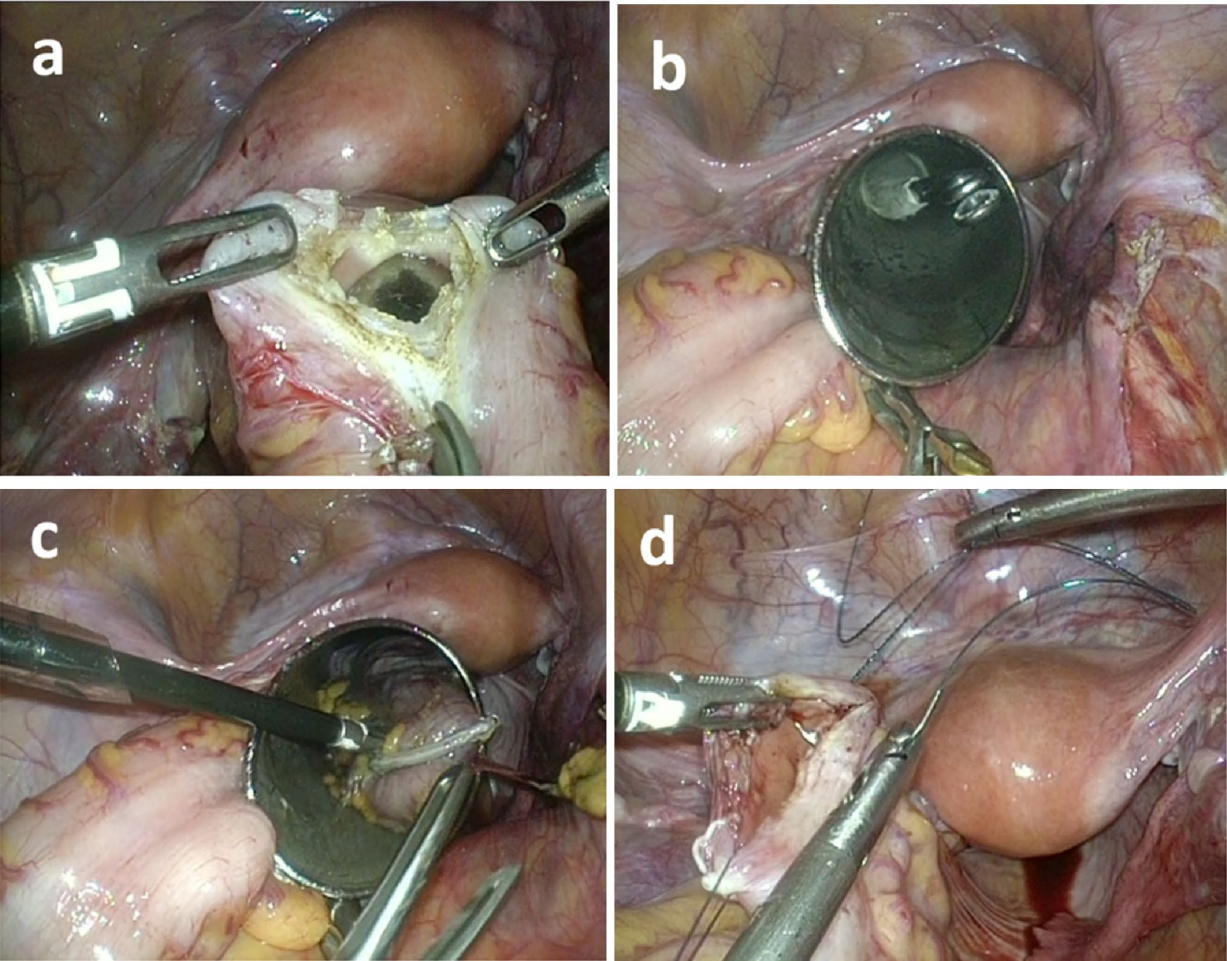
Surgical steps of transrectal NOSE approach. **a** Enterotomy made at the upper rectum. **b** Transanal endoscopic microsurgery (TEM) scope forwarded beyond the rectal opening, **c** Specimen pulled out through the TEM scope. **d** Rectal opening closed by barbed suture

For the conventional group with specimen extraction through a mini-laparotomy wound, the removal of a specimen was from the midline for EA, by extending the right lower quadrant port wound, or through a Pfannenstiel incision for IA. For the NOSE group (*n* = 25), the extraction of the specimen was either using the transvaginal (*n* = 2) or transrectal (*n* = 23) approach. The surgical steps of NOSE using the transrectal approach are shown in Fig. 1. The rectosigmoid colon lumen was blocked using a bowel clamp. After adequate rectal irrigation with povidone iodine water, the transanal endoscopic microsurgery (TEM) scope (Richard Wolf, Tubingen, Germany) was inserted through the anus and then gently pushed till it reached the upper rectum. An enterotomy was made at the upper rectum, using a suction device to clean any fecal spillage. The TEM scope was forwarded beyond the rectal opening, and then the specimen was pulled out through the TEM scope. The rectal opening was closed by barbed suture, and the air leak test was performed to identify mechanical failure. Two patients underwent right colectomy with transvaginal specimen extraction. The vagina was cleaned with povidone–iodine. The posterior vagina was opened and a double-ringed wound protector (Alexis wound retractor; Applied Medical, Rancho Santa Margarita, CA, USA) was used to protect and shorten the vaginal canal. Then the specimen was pulled out through the vaginal canal. The colpotomy incision was closed with 2-0 absorbable suture.

### Outcomes and covariables

Measurement outcomes included short-term postoperative complications, recovery, and readmission. Postoperative complications were defined as morbidity occurring within 30 days and included wound-related complications (wound infection or wound dehiscence); pulmonary (atelectasis or pneumonia), cardiovascular (myocardial infarction, stroke, or embolism), urinary (urinary tract infection or neurogenic bladder), gastrointestinal (obstruction, ileus, or bleeding), or abdominal (abscess or internal bleeding) complications; anastomosis (leakage or stenosis); and other rare complications. Postoperative mortality was defined as death occurring within 30 days after an operation. Postoperative recovery evaluation was based on blood test reports, time to first flatus and stool passage, time to diet, pain intensity, length of hospital stay. Postoperative 30-day hospital readmission data were also collected. For postoperative pain assessment, the patients were subdivided into two groups: the patient-controlled analgesia (PCA) group and the non-PCA group. Pain intensity was assessed using a Numeric Rating Scale (NRS) with scores from 0 to 10, (10 = the worst pain). The highest pain scores of patients on each day for 3 consecutive days postoperatively were used for further evaluation.

### Statistical analysis

All analyses were conducted using IBM SPSS Statistics, Version 21.0 (Armonk, NY, USA: IBM Corp.). Clinicopathological characteristics with categorical variables were presented as frequencies and proportions and were compared using the Chi-square test. Continuous variables were expressed as means and standard deviations and were analyzed using the Student’s *t* test. Statistical significance was set at *p* < 0.05.

## Results

We enrolled 297 patients (151 males, mean age 64.9 ± 12.8 years) who underwent minimally invasive right hemicolectomy. In these 297 patients, 272 patients had conventional surgery with specimen extraction through mini-laparotomy and 25 patients had NOSE surgery (23 transrectal, 2 transvaginal). The demographic data of these patients are presented in Table 1. The two groups did not differ significantly in terms of age, sex, BMI, presence of medical illness (including hypertension, cardiac disease, cardiovascular accident, asthma, diabetes mellitus, peptic ulcer, hepatitis, liver cirrhosis, gallstone disease, and thyroid problems), and preoperative laboratory data (hemoglobin levels, white blood cell [WBC] counts, percentages of segmented WBC, serum albumin levels, blood urea nitrogen levels, creatinine levels, aspartate aminotransferase levels, and total bilirubin levels). The NOSE group had a higher rate of robotic surgery (12.0% vs. 3.3%, *p* = 0.035) and a higher rate of intracorporeal anastomosis (100.0% vs. 52.6%, *p* < 0.001) than the conventional group. The diagnosis of malignant colon disease did not differ significantly between the two groups (malignancy rate in conventional vs. NOSE, 89.3% vs. 88.0%, *p* = 0.836). Among the patients with malignancy (conventional vs. NOSE, 243 vs. 22), the tumors were significantly larger in the conventional than in the NOSE group (conventional vs. NOSE, 4.4 ± 2.2 cm vs. 3.4 ± 1.6 cm, *p* = 0.007).

**Table 1:** Clinicopathological features of patients who underwent minimally invasive right hemicolectomy

	Conventional (272)	NOSE (25)	Missing data	*p*
Age (years)	65.3 ± 12.7	61.0 ± 13.4		0.105
Sex				0.766
Male	139 (51.1)	12 (48)		
Female	133 (48.9)	13 (52)		
BMI (kg/m^2^)			1	0.375
BMI ≦ 25	116 (42.8)	13 (52)		
BMI > 25	155 (57.2)	12 (48)		
BMI, mean	24.7 ± 4.2	25.2 ± 3.5		0.573
Medical illness				
Hypertension	124 (45.6)	9 (36)		0.356
Cardiac disease	25 (9.2)	1 (4)		0.379
CVA	7 (2.6)	1 (4)		0.673
Asthma	6 (2.2)	0		0.453
Diabetes	68 (25)	4 (16)		0.315
Peptic ulcer	24 (8.8)	1 (4)		0.406
Hepatitis	15 (5.5)	1 (4)		0.748
Liver cirrhosis	3 (1.1)	0		0.598
Cholelithiasis	5 (1.8)	0		0.494
Thyroid problem	9 (3.3)	1 (4)		0.855
Other	63 (23.2)	5 (20)		0.719
Lab data				
Hb (g/dL)	11.6 ± 2.6	12.4 ± 2.4		0.153
WBC (/uL)	7222 ± 2307	6532 ± 2008		0.149
Seg (%)	63.9 ± 9.8	61.5 ± 9.7	3	0.250
Albumin (g/dL)	4.1 ± 0.4	4.2 ± 0.4	1	0.555
BUN (mg/dL)	17.2 ± 9.4	15.8 ± 8.0	1	0.499
Cr (mg/dL)	1.0 ± 1.1	0.9 ± 0.4	2	0.474
AST (U/L)	25 ± 11	32 ± 34	4	0.309
Total bilirubin (mg/dL)	0.5 ± 0.4	0.6 ± 0.3		0.113
Technique				0.035
Laparoscopic	263 (96.7)	22 (88)		
Robotic	9 (3.3)	3 (12)		
IA	143 (52.6)	25 (100)		< 0.001
Diagnosis				0.836
Malignant	243 (89.3)	22 (88)		
Benign	29 (10.7)	3 (12)		
Malignancy	(*n* = 243)	(*n* = 22)		
Tumor size (cm)	4.4 ± 2.2	3.4 ± 1.6	1	0.007
CEA (ng/mL)				0.381
CEA < 5	178 (73.3)	18 (81.8)		
CEA > 5	65 (26.7)	4 (18.2)		

Values are presented as mean ± standard deviation or number (%)

*NOSE* natural orifice specimen extraction, *BMI* body mass index, *CVA* cerebrovascular accident; *Hb* hemoglobin, *WBC* white blood cells, *Seg* segmented neutrophils, *BUN* blood urea nitrogen, *Cr* creatinine, *AST* aspartate aminotransferase, *IA* intracorporeal anastomosis, *CEA* carcinoembryonic antigen

The postoperative short-term outcomes are listed in Table 2. The operating time did not differ significantly between the two groups (conventional vs. NOSE, 248.0 ± 78.3 min vs. 247.8 ± 84.4 min, *p* = 0.988). Furthermore, the blood loss during surgery did not differ significantly between the two groups. The rates of surgery combined with resection of other involved organs were similar in these two groups (conventional vs. NOSE, 9.6% vs. 8.0%, *p* = 0.799). The overall postoperative morbidity rate did not differ significantly between the two groups although the conventional group had a higher morbidity rate than the NOSE group (conventional vs. NOSE, 12.9% vs. 4.0%, *p* = 0.194). In the subgroup of postoperative complications, the two groups did not differ significantly in any of the postoperative variables (wound, pulmonary, cardiovascular, urinary, gastrointestinal, abdominal, and anastomosis). There was no deep or organ space surgical site infection in the NOSE group. None of the patients complained about anal bleeding, anal pain, and fecal or gas incontinence after the NOSE procedure. The reoperation rate and readmission rate did not differ significantly between the two groups. Postoperative mortality rates were comparable (*p* = 0.761) in the conventional group (1 patient, 0.4%) and NOSE group (0 patient). The mean follow-up time of the NOSE group was 13.36 months (range 1–25) months. None of the patients in the NOSE group had rectal tumor seeding during the follow-up period.

**Table 2:** Perioperative outcomes

	Conventional (272)	NOSE (25)	*p*
Operation time (minutes)	248.0 ± 78.3	247.8 ± 84.4	0.988
Blood loss (mL)	45 ± 49	32 ± 15	0.185
Combined surgery	26 (9.6)	2 (8)	0.799
Postoperative morbidity	35 (12.9)	1 (4)	0.194
Wound	5 (1.8)	0	0.494
Pulmonary	1 (0.4)	0	0.761
Cardiovascular	1 (0.4)	0	0.761
Urinary	1 (0.4)	0	0.761
Gastrointestinal	14 (5.1)	1 (4)	0.802
Abdominal	7 (2.6)	0	0.417
Anastomosis	6 (2.2)	0	0.453
Mortality	1 (0.4)	0	0.761
Second operation	6 (2.2)	0	0.453
Re-admission	6 (2.2)	0	0.453

Values are presented as mean ± standard deviation or number (%)

*NOSE* natural orifice specimen extraction, *DVT* deep vein thrombosis

Postoperative clinical information is presented in Table 3. Laboratory data checked on postoperative day (POD) 3 did not exhibit significant differences in WBC counts, percentages of segmented WBC, and C-reactive protein (CRP) levels between the two groups. The time of first flatus was comparable in the two groups (POD 1.8 ± 0.7 vs. POD 2.4 ± 1.4, *p* = 0.066). The NOSE group had earlier bowel movements (POD 3.0 ± 1.2 vs. POD 4.2 ± 2.0, *p* < 0.001), tolerance to liquid diet (POD 2.6 ± 1.1 vs. POD 4.3 ± 2.9, *p* = 0.004) and tolerance to soft diet (POD 4.5 ± 2.5 vs. POD 6.1 ± 3.3, *p* = 0.020) than the conventional group. The postoperative hospital stay was significantly shorter in the NOSE group (mean 5.2 ± 2.8 days, median 5 days, range 3–17 days) than in the conventional group (mean 8.3 ± 5.1 days, median 7 days, range 3–45 days) (*p* = 0.004).

**Table 3:** Postoperative laboratory data and recovery parameters

	Conventional (272)	NOSE (25)	*p*
POD3 lab data			
WBC (/uL)	9606 ± 2959 (*n* = 245)	10,264 ± 2327 (*n* = 22)	0.312
Seg (%)	77.3 ± 7.2 (*n* = 245)	79.4 ± 7.5 (*n* = 22)	0.177
CRP (mg/L)	82.5 ± 52.2 (*n* = 245)	72.4 ± 36.6 (*n* = 22)	0.379
First flatus passage (days)	2.4 ± 1.4	1.8 ± 0.7	0.066
First stool passage (days)	4.2 ± 2.0	3.0 ± 1.2	< 0.001
Tolerate liquid diet (days)	4.3 ± 2.9	2.6 ± 1.1	0.004
Tolerate soft diet (days)	6.1 ± 3.3	4.5 ± 2.5	0.020
Mean postoperative hospital stay (days)	8.3 ± 5.1	5.2 ± 2.8	0.004
Median postoperative hospital stay (days)	7 (3–45)	5 (3–17)	

Values are presented as mean ± standard deviation unless otherwise indicated

*NOSE* natural orifice specimen extraction, *POD* postoperative day, *WBC* white blood cells, *Seg* segmented neutrophils, *CRP* C-reactive protein

**Fig. 2:**
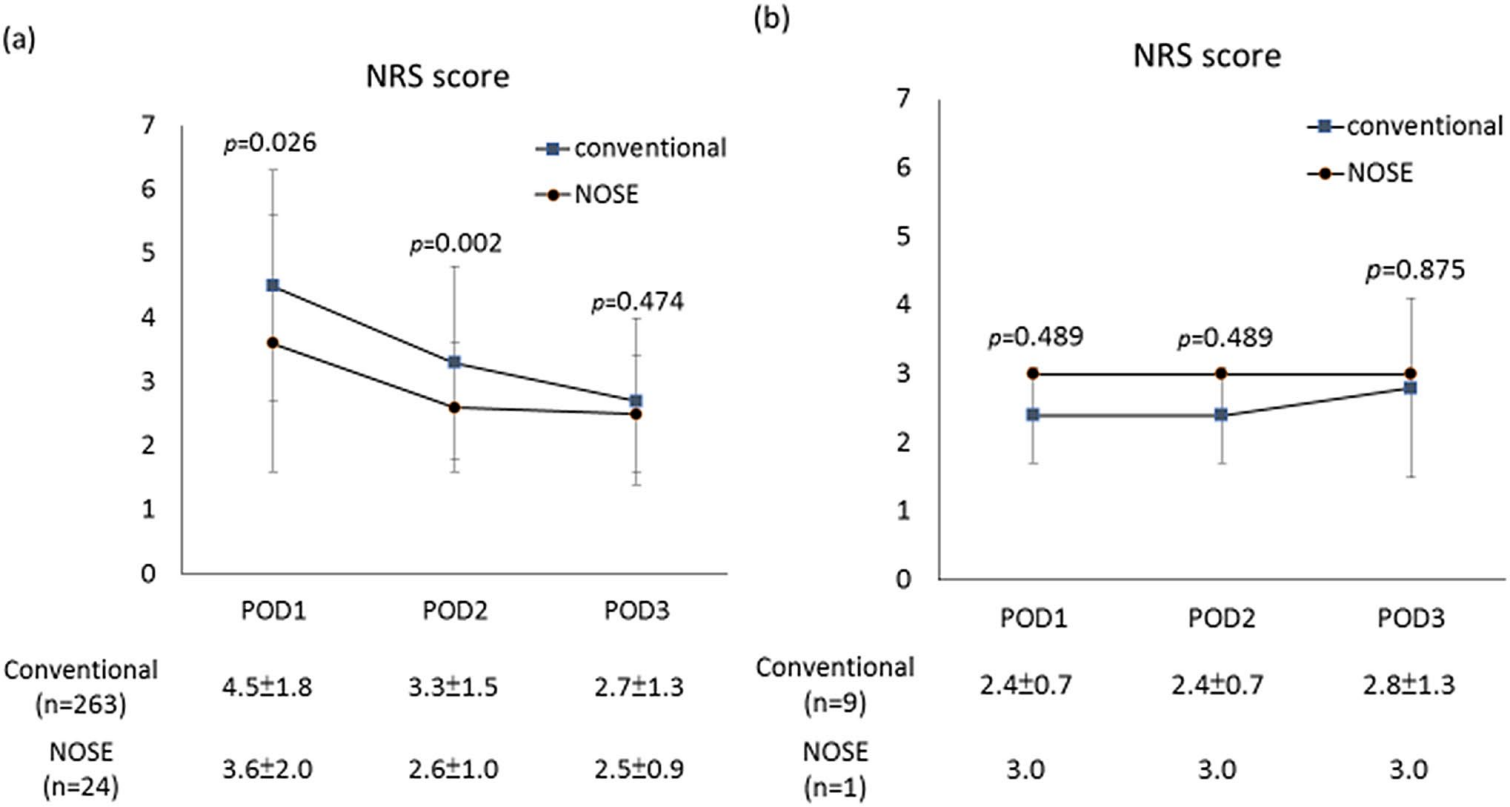
Postoperative pain scores in patients without patient-controlled analgesia (**a**) and patients with patient-controlled analgesia (**b**). Values are presented as mean ± standard deviation. *NRS* Numeric Rating Scale, *NOSE* natural orifice specimen extraction, *POD* postoperative day

Figure 2 shows the difference in scores on the NRS on 3 consecutive days after operation in the two groups. The NRS scores of patients without PCA (conventional 263, NOSE 24) are shown in Fig. 2a and the scores of the patients with PCA (conventional 9, NOSE 1) are shown in Fig. 2b.The conventional group exhibited significantly higher NRS scores on POD1 and POD2 than the NOSE group (POD1, conventional vs. NOSE: 4.5 ± 1.8 vs. 3.6 ± 2.0, *p* = 0.026; POD2, conventional vs. NOSE: 3.3 ± 1.5 vs. 2.6 ± 1.0, p = 0.002) (Fig. 2a). The NRS did not differ in POD1-3 between the two groups (Fig. 2b).

## Discussion

Minimally invasive surgery for colorectal disease has been widely accepted; this surgery causes smaller abdominal wounds than open surgery. However, a mini-laparotomy wound is necessary to remove specimen. To reduce wound-related complications and achieve superior cosmetic results, the NOSE procedure was developed. The advantages and disadvantages of conventional and NOSE surgery for right colectomy are summarized in Table 4. Some studies have reported that laparoscopic right hemicolectomy with transvaginal specimen retrieval is feasible with favorable short-term surgical outcomes [16–18]. Transanal extraction of the sigmoid colon and rectum specimens also has acceptable short-term and long-term outcomes [11, 13]. In this study, we mainly performed minimally invasive right hemicolectomy using the NOSE method to extract the specimen transrectally. The transvaginal method can also be used in the NOSE procedure; however, colpotomy incision repair is more difficult than rectal repair. An incision was made longitudinally over the upper rectum, and suture repair was quite simple. In addition, transrectal specimen removal can be performed in male and female patients and can avoid adverse effects on sexual function. Some researchers have expressed concern about the bacteriological safety of the transrectal method because the rectum is opened for specimen retrieval. We observed that no significant postoperative morbidity and changes in laboratory data are observed if the rectum is cleaned properly. To our knowledge, our study is the first evaluating short-term surgical outcomes of minimally invasive right hemicolectomy performed using transrectal NOSE.

**Table 4:** Summary and comparison of conventional and NOSE surgery for right colectomy

Variable	Conventional	NOSE
Wound size	Larger	Smaller
Wound pain	More	Less
Anastomosis leakage	Equal	Equal
Intraabdominal abscess	Equal	Equal
Rectal complication	No	Potential
Bowel recovery	Slower	Faster
Hospital stay	Longer	Shorter
Specimen size restriction	No	Yes
Intracorporeal suture technique	Optional	Required
Wound-related complications	More	Less

*NOSE* natural orifice specimen extraction

Avoidance of the mini-laparotomy wound for specimen extraction is one of the most crucial features of NOSE. Compared with conventional minimally invasive surgery, NOSE causes less postoperative pain, results in faster recovery, and provides superior cosmetic results. A previous study demonstrated favorable short-term surgical outcomes after performing laparoscopic right hemicolectomy using the transvaginal NOSE [18]. Patients who had undergone NOSE experienced less pain and required shorter hospital stays than those who had undergone conventional surgery, without significant differences in surgical morbidity. Two randomized clinical trials that compared the short-term operative outcomes in patients with left-sided colonic disease [19, 20]. They demonstrated that NOSE group experienced less wound pain and had a lower wound infection rate than the conventional group. In our study, no significant differences were observed in the operation time, blood loss, and postoperative morbidity between the two groups. The postoperative pain scores did not differ significantly in the two groups among patients with PCA. As regards patients without PCA, the NOSE group experienced less wound pain than the conventional group. The benefit of NOSE was observed on POD 1 and POD2. Previous studies have reported time to flatus passage of approximately 2.7–3 days and time to resumption of a regular diet of approximately 4–4.6 days for patients who had undergone transvaginal NOSE [17, 18]. Faster bowel recovery and earlier food intake were observed in the NOSE group than in the conventional group. Early ambulation and less use of analgesic agents because of less postoperative pain may be the reasons. This can help to shorten the hospital stay. Surgical site infection occurs after conventional laparoscopic right hemicolectomy in 5–7% of cases [21, 22]. None of our patients who had NOSE experienced wound-related complications.

Bacterial contamination is always a concern during the NOSE procedure. We strongly suggest that mechanical bowel preparation, intraoperative transanal lavage with povidone iodine solution, transluminal wound protector, and prophylactic antibiotics are applied to reduce the bacterial load [23]. Recently, a study showed that the risk of bacterial contamination with NOSE was not significantly higher than that in conventional laparoscopic surgery [24]. In our study, patients who had NOSE did not experience significant postoperative morbidity or laboratory data changes, such as leukocytosis, or CRP level elevation, than the conventional group. None of our patients have had rectal wound-related complications or leakage thus far.

Tumor size is considered before applying the NOSE procedure. Many authors limit indications to tumors smaller than 3 [25, 26], 4 [27, 28], 5 [29, 30], 6 [31], or 6.5 [32] cm. The average tumor size in the NOSE group in our study was 3.4 cm, and it was significantly smaller than that in the conventional group. Some authors have stated that obese patients are not suitable for transrectal specimen extraction and set the BMI cutoff at > 28 kg/m^2^ [32], > 30 kg/m^2^ [29], or > 35 kg/m^2^ [33]. In our study, although no significant difference in patients’ BMI was seen in the two groups, the highest BMI was 32 kg/m^2^ in the NOSE group and 40 kg/m^2^ in the conventional group. Patients with a bulky mesocolon, a narrow pelvis, and previous pelvic surgery with severe adhesions were not eligible for NOSE.

This study has some limitations. First, the retrospective analysis of prospectively collected data might have caused some selection bias. Notably, the NOSE group is a highly selective patient group, which is not comparable to the conventional group. Second, the sample size is relatively small in the NOSE group, which might have resulted in a lack of statistical power. Third, this study only reports on short-term outcomes and lacks long-term oncologic outcome follow-up; however, the use of the NOSE procedure in the left colon is well established.

## Conclusions

Transrectal NOSE can be performed in some patients who require minimally invasive right hemicolectomy with postoperative short-term outcomes that are comparable to those of conventional laparoscopic surgery. NOSE is associated with less postoperative wound pain, faster bowel recovery, and shorter hospital stay than the conventional method. Additional prospective studies with larger patient populations and longer follow-up are warranted.
